# Spatial distribution and habitat suitability of *Biomphalaria straminea*, intermediate host of *Schistosoma mansoni,* in Guangdong, China

**DOI:** 10.1186/s40249-018-0492-6

**Published:** 2018-11-05

**Authors:** Ya Yang, Shao-Yu Huang, Fu-Quan Pei, Yue Chen, Qing-Wu Jiang, Zhuo-Hui Deng, Yi-Biao Zhou

**Affiliations:** 1 Key Laboratory of Public Health Safety, Ministry of Education, Tropical Disease Research Center, Department of Epidemiology, School of Public Health, Fudan University, Shanghai, China; 2 Guangdong Provincial Center for Disease Control and Prevention, WHO Collaborating Centre for Surveillance, Research and Training of Emerging Infectious Diseases, Guangzhou, Guangdong China; 3 School of Epidemiology and Public Health, Faculty of Medicine, University of Ottawa, Ottawa, Canada

**Keywords:** *Biomphalaria straminea*, Distribution, Environmental factor, Guangdong, China

## Abstract

**Background:**

*Biomphalaria straminea* is an invasive vector in China, posing a significant threat to public health. Understanding the factors affecting the establishment of this snail is crucial to improve our ability to manage its dispersal and potential risk of schistosomiasis transmission. This study sought to determine the spatial distribution of *B. straminea* in mainland China and whether environmental factors were divergent between places with and without *B. straminea*.

**Methods:**

A malacological survey of *B. straminea* was conducted in Guangdong Province, China. Snails were identified using anatomical keys. Water and sediment samples were taken, and their physicochemical properties were analyzed using national standard methods. Landscape and climatic variables were also collected for each site. We compared the environmental characteristics between sites with and without *B. straminea* using Mann-Whitney U test. We further used generalized linear mixed models to account for seasonal effects.

**Results:**

*B. straminea* was found at six sites, including one in Dongguan and five in Shenzhen. Probability map found a hot spot of *B. straminea* distribution at Shenzhen and Hong Kong. Sites occupied by *B. straminea* were characterized by higher median altitude, mean annual precipitation and moderate temperature. Water with snails had higher median concentrations of total nitrogen, nitrate and nitrites, ammoniacal nitrogen, calcium, zinc and manganese but lower dissolved oxygen and magnesium. Sediments with snails had higher median copper, zinc and manganese. *B. straminea* was associated with maximum temperature of the warmest month (pMCMC < 0.001) and sediment zinc (pMCMC < 0.001).

**Conclusions:**

*B. straminea* is distributed in Shenzhen and its surrounding areas in Guangdong, China. Sites with and without *B. straminea* differed in the maximum temperature of the warmest month and sediment zinc. Surveillance should be continued to monitor the dispersal of this snail in China.

**Electronic supplementary material:**

The online version of this article (10.1186/s40249-018-0492-6) contains supplementary material, which is available to authorized users.

## Multilingual Abstract

Please see Additional file 1 for translations of the Abstract into the five official working languages of the United Nations.

## Background

Schistosomiasis is one of the most widespread human parasitic diseases, affecting more than 200 million people worldwide [1, 2]. *Schistosoma mansoni*, whose intermediate hosts are freshwater snails of the genus *Biomphalaria*, is estimated to infect more than 80 million people in the tropical and subtropical areas of Africa, the Middle East, and South America [3]. Although *S. mansoni* is not currently endemic in China, imported schistosomiasis cases of such kind have been continuously reported in laborers returned from Africa [4].

The transmission of schistosomiasis is determined by the existence and geographic distribution of its host snails [5]. *Biomphalaria straminea* is an intermediate host of *S. mansoni* and is originated in southeastern South America [6]. *B. straminea* has gradually expanded its habitats to other states of Brazil and surrounding countries, which are attributed to its capacity to endure long periods of drought and greater fertility [3, 4]. In addition to the above peripheral range extensions, *B. straminea* is known for intercontinental dispersal to Hong Kong of China in 1974, possibly through tropical aquarium plants or fish trade with South America [7]. Since then, the snail has been found in different water habitats in Hong Kong and Shenzhen, Dongguan, and Huizhou of Guangdong Province, China [3, 8].

In the context of globalization, especially with the advent of China’s Belt and Road Initiative [9], growing movement of goods and people could increase the risk of transmission of *S. mansoni* in China in the presence of *B. straminea* snails. Therefore, there was an urgent need to determine the spatial distribution of *B. straminea* in China. *B. straminea* has established in various freshwater habitats in Hong Kong but no evidence of *S. mansoni* was found in the snails [10]. However, there is currently a lack of large-scale, comprehensive snail survey in mainland China. Moreover, previous studies have shown that environmental and ecological factors, including temperature, light, water chemistry and conductivity and aquatic vegetation, can influence the distribution and abundance of *Biomphalaria* snails [6, 11]. The purpose of this study was to determine the spatial distribution of *B. straminea* in mainland China and whether environmental factors differ between places with and without *B. straminea*.

## Methods

### Study area

Guangdong Province, located in southeast China, consists of twenty-one prefecture-level divisions. This region experiences a subtropical monsoon climate, featuring hot and humid summers, and mild winters. The averages of annual temperature and annual precipitation are 21.8 °C and 1789.3 mm, respectively. Guangdong has extended water systems. Generally, these rivers can be divided into the Pearl River (including three upstream rivers: the East River, North River, and West River), the Han River and other minor rivers along the coast.

### Study design

**Fig. 1:**
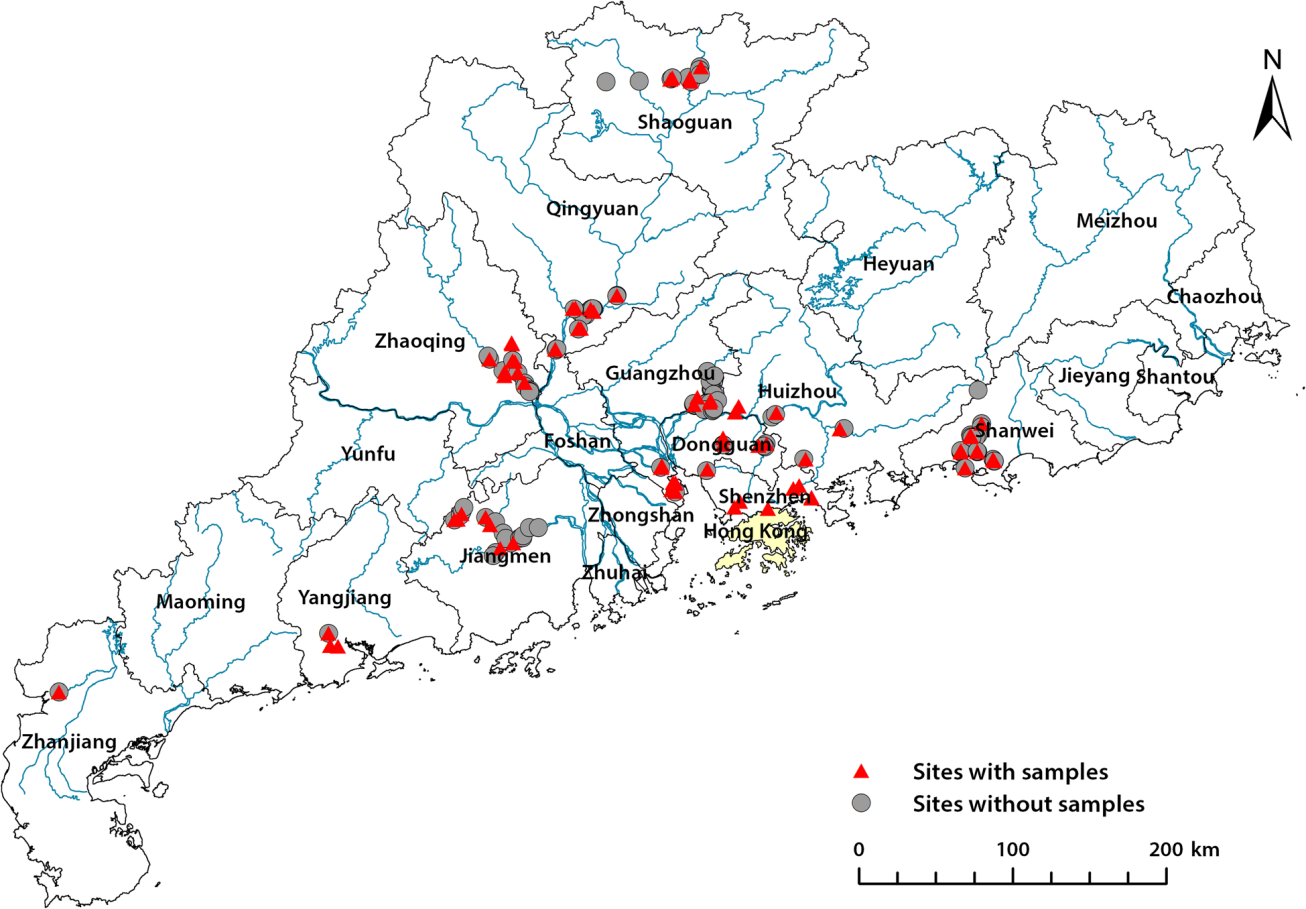
Geographical location of surveyed sites in the study region. Gray circles indicates sites without water or sediment samples, and red triangles represents those with samples

One hundred and eighty-seven study sites were from 11 municipals (Shenzhen, Zhanjiang, Yangjiang, Jiangmen, Guangzhou, Dongguan, Huizhou, Shanwei, Zhaoqing, Qingyuan, and Shaoguan) of Guangdong Province (see Fig. 1). These sites were selected based on the presence of aquatic habitats, accessibility along the Pearl River and its tributaries and existence of *B. straminea* reported by previous studies. Swamps and puddles adjacent to the river and streams were also sampled. Given the considerable area of Guangdong, the sampling events carried out during November 2016 to October 2017. Each site was surveyed once at approximately the same time of day, in order to make comparisons. The sampling sites were coded based on location and point of sampling. The geographic coordinates of each site were recorded with a handheld Global Positioning System (GPS) device (Trimble Navigation Inc., Sunnyvale, USA).

### Snail sampling

Predefined protocols were used for the snail survey [12]. In brief, sampling was carried out by two trained field investigators with a scoop. At each site, the permitted sampling time was 30 min. Any snail in a radius of approximately 2 m was captured. All collected snails were labeled, transferred to the laboratory and identified using anatomical keys including the shape of shells and the number of prostate diverticula [6].

### Water and sediment analysis

The samples were collected on sunny or cloudy days to minimize the effects of weather conditions. Surface water samples were collected at 20 cm depth using polyethylene plastic bottles. The bottles were triple rinsed with sample water prior to collection. Surface sediments (0–10 cm) were collected at the edge of the bank, where the snails are most likely to be present, using a plastic shovel. Sediments were stored in pre-rinsed polyethylene plastic bottles. Water and sediment samples were clearly labeled with the location name. GPS coordinates, time and date of survey. Water and sediment samples were kept refrigerated and delivered to the laboratory within 2–5 days of collection. Water samples were measured for pH, electrical conductivity (EC) and the concentrations of total nitrogen (TN), nitrate and nitrites (NO_x-_), ammonia nitrogen (NH_3−_N), total phosphorus (TP), chemical oxygen demand (COD), dissolved oxygen (DO), calcium (Ca), magnesium (Mg), copper (Cu), zinc (Zn), iron (Fe), manganese (Mn), cadmium (Cd), lead (Pb), chromium (Cr) and nickel (Ni). Sediments were measured for pH, EC, total organic carbon (TOC) and the concentrations of Cu, Zn, Fe, Mn, Cd, Pb, Cr, and Ni. The detailed methods, instruments, and limits of detection (LOD) of each analysis can be found in the supplementary material (Additional file 2).

### Landscape and climatic data

We used mean annual precipitation (MAP, mm), mean annual temperature (MAT, °C), maximum temperature of warmest month (MaxTWM, °C), minimum temperature of coldest month (MinTCM, °C), mean temperature of warmest quarter (MTWQ, °C) and mean temperature of coldest quarter (MTCQ, °C) from WorldClim version 2.0, which uses historic global meteorological station data from 1970 to 2000 to interpolate global climate surfaces [13]. The spatial resolution of this climate surface is 30 s (approximately 1 km^2^). We extracted the variables for each sampling site according to the latitude and longitude.

We extracted the altitude of each site from the 30 m Shuttle Radar Topography Mission (SRTM) data. To analyze the association between the presence of *B. straminea* and vegetation canopy cover, we used mean annual normalized difference vegetation index (NDVI) values, extracted from the 1 km × 1 km resolution SPOT-VEGETATION NDVI layers for years 2011 through 2015. This mean NDVI product is a proxy for the level of living green plant canopies over a year. The 30 m SRTM and NDVI data sets are from by Data Center for Resources and Environmental Sciences, Chinese Academy of Sciences (RESDC) (http://www.resdc.cn).

### Statistical analysis

We calculated descriptive statistics for environmental variables. The relationships among variables were analyzed using Spearman’s rank correlations test and were visualized using corrplot package [14]. The Mann-Whitney U test was used to test the heterogeneity of environmental and physicochemical characteristics for sites with and without *B. straminea*.

We first estimated the relationship between the presence of *B. straminea* (as a binary response variable) and environmental and physicochemical variables by fitting generalized linear mixed model (GLMM). The models were limited to variables with at least 50% of concentrations above LOD. Measurements below LODs were assigned one-half of the LOD values. Normality of variables was determined with visual inspection of data, and log10 transformation was used when necessary. Site code and survey month were used as random effects to account for the seasonal effects on the measurements and between site variability. The GLMM was built using Markov Chain Monte Carlo (MCMC) in the R package MCMCglmm [15]. For each model, the MCMC chains were run for 50 000 iterations with a burn-in of 10 000 and thinning interval of 20, to get posterior sample sizes of 2000. We fitted models that included significant variables from the univariate GLMMs and used backward selection to identify the minimal adequate model that retained only significant variables. All pairwise correlations between the included predictors were lesser than 0.40. We summarized parameter estimates using posterior means and 95% credible intervals (*CI*). A significance level of 0.05 was used for all tests. All analyses were done using R software (version 3.4.1, The R Project for Statistical Computing, https://www.r-project.org/).

We obtained additional occurrence data of *B. straminea* from a recent survey in Hong Kong during 2016–2017, which used similar snail sampling and identification methods to our study [10]. These presence points, together with data of our own survey, were spatially interpolated using Empirical Bayesian Kriging (EBK) in ArcGIS 10.2.2 (Environmental Systems Research Institute, Inc., Redlands, USA) to predict the spatial distribution of *B. straminea* in China [16]. Default general settings were used in the process of EBK.

## Results

### Spatial distribution of *B. straminea*

**Fig. 2:**
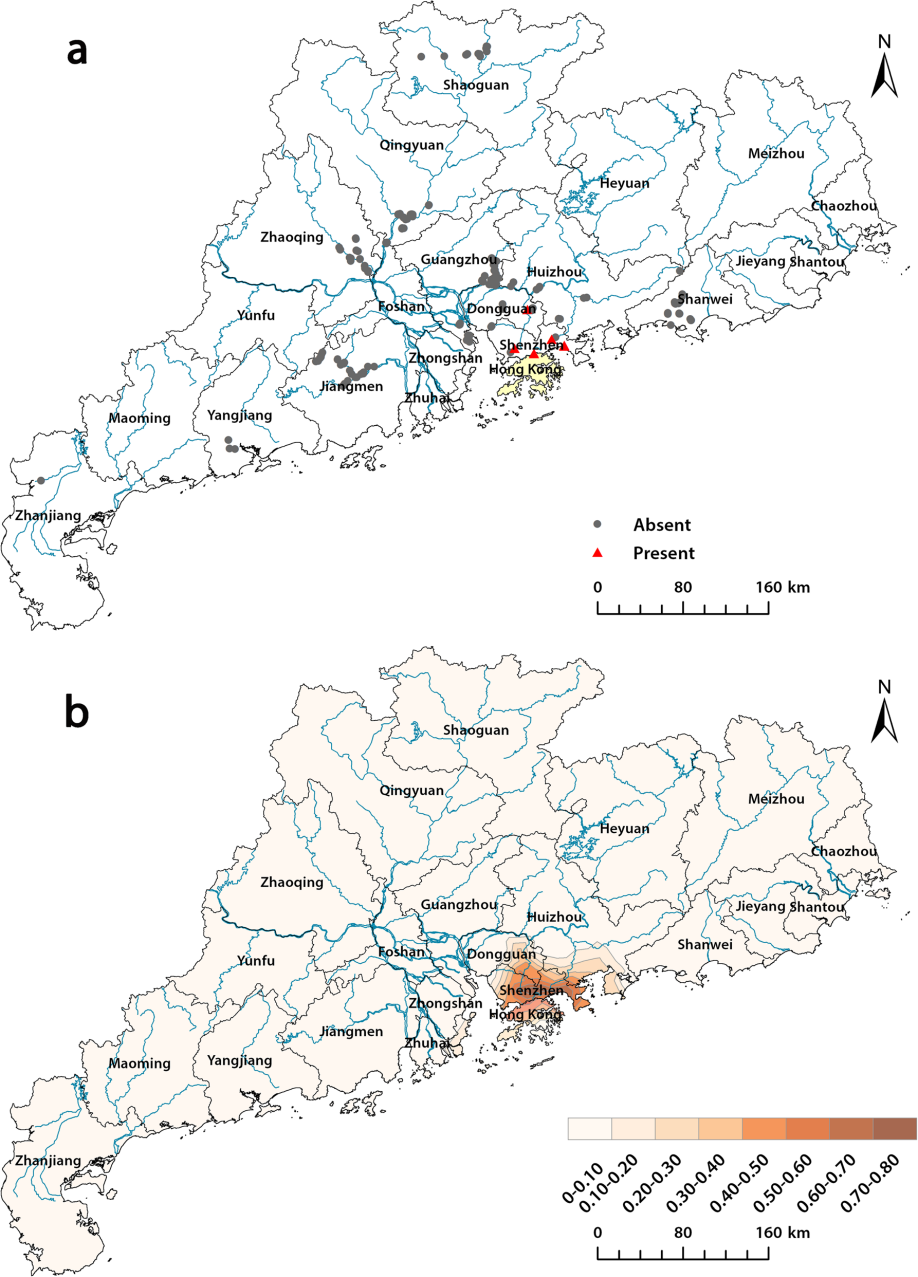
Geographical distribution of the survey sites (**a**) and probability map of the *B. straminea* snails in China (**b**). Probability map was built using presence data from our study and a survey in Hong Kong

*Biomphalaria straminea* was found at 6 sites, including 1 in Dongguan and 5 in Shenzhen during 2016–2017 (Fig. 2a). A hot spot for the distribution of *B. straminea* was found at the areas around Shenzhen and Hong Kong (Fig. 2b).

### Environmental and physicochemical characteristics

The altitude of sites ranged from minus 8 m to 190 m (Table 1). Most of the sites were well vegetated, with NDVI values over 0.6. Mean annual precipitation ranged from 1485.0 mm to 2106.0 mm, and the mean annual temperature showed values from 19.1 °C to 23.1 °C. Other temperature-related variables are also summarized in Table 1.

**Table 1:** Distributions of landscape and climatic characteristics of surveyed sites

Variables	Min	25%	50%	75%	Max	Mean	SD	CV
Altitude (m)	−8.0	2.0	9.0	36.0	190.0	26.2	39.7	1.52
NDVI	0.2	0.6	0.7	0.8	0.9	0.6	0.1	0.22
Mean annual precipitation (MAP, mm)	1485.0	1695.0	1718.0	1845.0	2106.0	1771.4	156.2	0.09
Mean annual temperature (MAT, °C)	19.1	22.0	22.2	22.4	23.1	22.1	0.7	0.03
Maximum temperature of warmest month (MaxTWM, °C)	31.5	32.6	33.1	33.7	34.2	33.1	0.7	0.02
Minimum temperature of coldest month (MinTCM, °C)	5.0	9.9	10.7	11.7	12.8	10.6	1.6	0.15
Mean temperature of warmest quarter (MTWQ, °C)	27.2	28.2	28.5	28.6	28.8	28.4	0.3	0.01
Mean temperature of coldest quarter (MTCQ, °C)	9.8	14.3	14.9	15.3	16.3	14.6	1.3	0.09

*SD* Standard deviation, *CV* Coefficient of variation, *NDVI* normalized difference vegetation index

The water and sediment chemistry properties are shown in Table 2. Water pH values varied from 4.91 to 7.78. The mean concentrations of TN, NO_x_^−^ and NH_3−_N were 10.40 mg/L, 1.66 mg/L, and 1.11 mg/L, respectively, with NH_3−_N showing the highest variability. The TP concentration ranged from 0.01 mg/L (< LOD) to 3.47 mg/L. The mean COD and DO concentrations were 14.59 mg/L and 5.25 mg/L. Electrical conductivity ranged from 68.85 mS/m to 1677 mS/m. The average Ca, Mg, Zn, Fe and Mn concentrations were 18.51 mg/L, 1.10 mg/L, 0.14 mg/L, 0.81 mg/L and 0.27 mg/L, respectively. Over half Cu (54.1%) and Ni (73.8%) measurements were below LODs. All water Cd, Pb, and Cr were below LODs.

**Table 2:** Distributions of measured water and sediment parameters in sampled sites

	*N*	*n*(%) < LOD	Min	25%	50%	75%	Max	Mean	SD	CV
Water variables										
pH	84	0 (0)	6.69	4.91	6.48	6.72	7.02	7.78	0.58	0.07
Total nitrogen (TN, mg/L)	84	0 (0)	0.31	1.29	2.72	9.05	62.38	10.40	16.54	1.59
Nitrate and nitrites (NO_x_^−^, mg/L)	84	1 (1.2)	< LOD	0.32	0.81	1.9	12.22	1.66	2.38	1.43
Ammoniacal nitrogen (NH_3−_N, mg/L)	84	16 (19)	< LOD	0.10	0.38	1.12	10.81	1.11	2.03	1.83
Total phosphorus (TP, mg/L)	84	1 (1.2)	< LOD	0.07	0.14	0.24	3.47	0.25	0.46	1.84
Chemical oxygen demand (COD, mg/L)	84	10 (11.9)	< LOD	4.94	10.18	21.71	62.75	14.59	13.07	0.9
Dissolved oxygen (DO, mg/L)	84	0 (0)	0.01	4.63	5.53	6.37	8.51	5.25	1.75	0.33
Electrical conductivity (EC, mS/m)	84	0 (0)	8.47	68.85	135.95	224.75	1677	199.63	266.31	1.33
Calcium (Ca, mg/L)	84	2 (2.4)	< LOD	1.18	5.04	21.78	202.51	18.51	35.58	1.92
Magnesium (Mg, mg/L)	84	4 (4.8)	< LOD	0.66	1.10	1.53	3.15	1.10	0.65	0.59
Copper (Cu, mg/L)	74	40 (54.1)	< LOD	< LOD	< LOD	0.05	0.1	0.04	0.02	0.56
Zinc (Zn, mg/L)	74	27 (36.5)	< LOD	< LOD	0.01	0.05	1.52	0.14	0.32	2.29
Iron (Fe, mg/L)	56	6 (10.7)	< LOD	0.17	0.44	1.02	5.34	0.81	1.01	1.25
Manganese (Mn, mg/L)	74	2 (2.7)	< LOD	0.05	0.14	0.27	1.86	0.27	0.37	1.37
Cadmium (Cd, mg/L)	84	84 (100)	< LOD	< LOD	< LOD	< LOD	< LOD	–	–	–
Lead (Pb, mg/L)	84	84 (100)	< LOD	< LOD	< LOD	< LOD	< LOD	–	–	–
Chromium (Cr, mg/L)	84	84 (100)	< LOD	< LOD	< LOD	< LOD	< LOD	–	–	–
Nickel (Ni, mg/L)	84	62 (73.8)	< LOD	< LOD	< LOD	0.03	0.12	0.03	0.03	1.14
Sediment variables										
pH	74	0 (0)	3.94	6.64	6.97	7.31	8.28	6.84	0.7	0.1
Electrical conductivity (EC, mS/m)	74	0 (0)	27.65	217.6	435	857.83	4685.8	676.29	817.11	1.21
Total organic carbon (TOC, g/kg)	75	0 (0)	16.22	20.29	27.41	38.21	71.08	30.33	11.62	0.38
Copper (Cu, mg/Kg)	71	15 (21.1)	< LOD	0.13	1.04	3.28	107.27	4.68	13.82	2.95
Zinc (Zn, mg/kg)	71	20 (28.2)	< LOD	< LOD	7.57	25.73	93.79	16.52	20.23	1.22
Iron (Fe, mg/L)	64	21 (32.8)	< LOD	< LOD	298.54	340.8	675.12	260.63	184.35	0.71
Manganese (Mn, mg/kg)	71	10 (14.1)	< LOD	0.6	75.83	139.75	600.8	87.05	92.78	1.07
Cadmium (Cd, mg/kg)	75	22 (29.3)	< LOD	< LOD	0.12	0.19	3.61	0.23	0.51	2.22
Lead (Pb, mg/kg)	75	5 (6.7)	< LOD	0.04	1.92	8.00	72.34	6.50	12.51	1.92
Chromium (Cr, mg/kg)	75	73 (97.3)	< LOD	< LOD	< LOD	< LOD	4.69	–	–	–
Nickel (Ni, mg/kg)	75	56 (74.7)	< LOD	< LOD	< LOD	1.98	34.37	2.76	4.44	1.61

*LOD* Limit of detection; All values below LOD were assigned a value equal to the LOD divided by 2

*N* Number of samples, *n*: Number of samples < LOD; *SD* Standard deviation, *CV* Coefficient of variation, *NDVI* normalized difference vegetation index; *Min* Minimum, *Max* Maximum

The pH for sediments was from 3.94 to 8.28. The average TOC was 30.33 g/Kg. The average Cu, Zn, Fe, Mn, Cd and Pb concentrations in sediment were 4.46 mg/kg, 16.52 mg/kg, 260.63 mg/L, 87.059 mg/kg, 0.23 mg/kg, 6.50 mg/kg and 3.17 mg/kg, respectively. Ni measurements varied from < LOD to 34.37 mg/kg. 97.3% of sediment samples had Cr concentration below LOD.

### Relationships among environmental and physicochemical variables

**Fig. 3:**
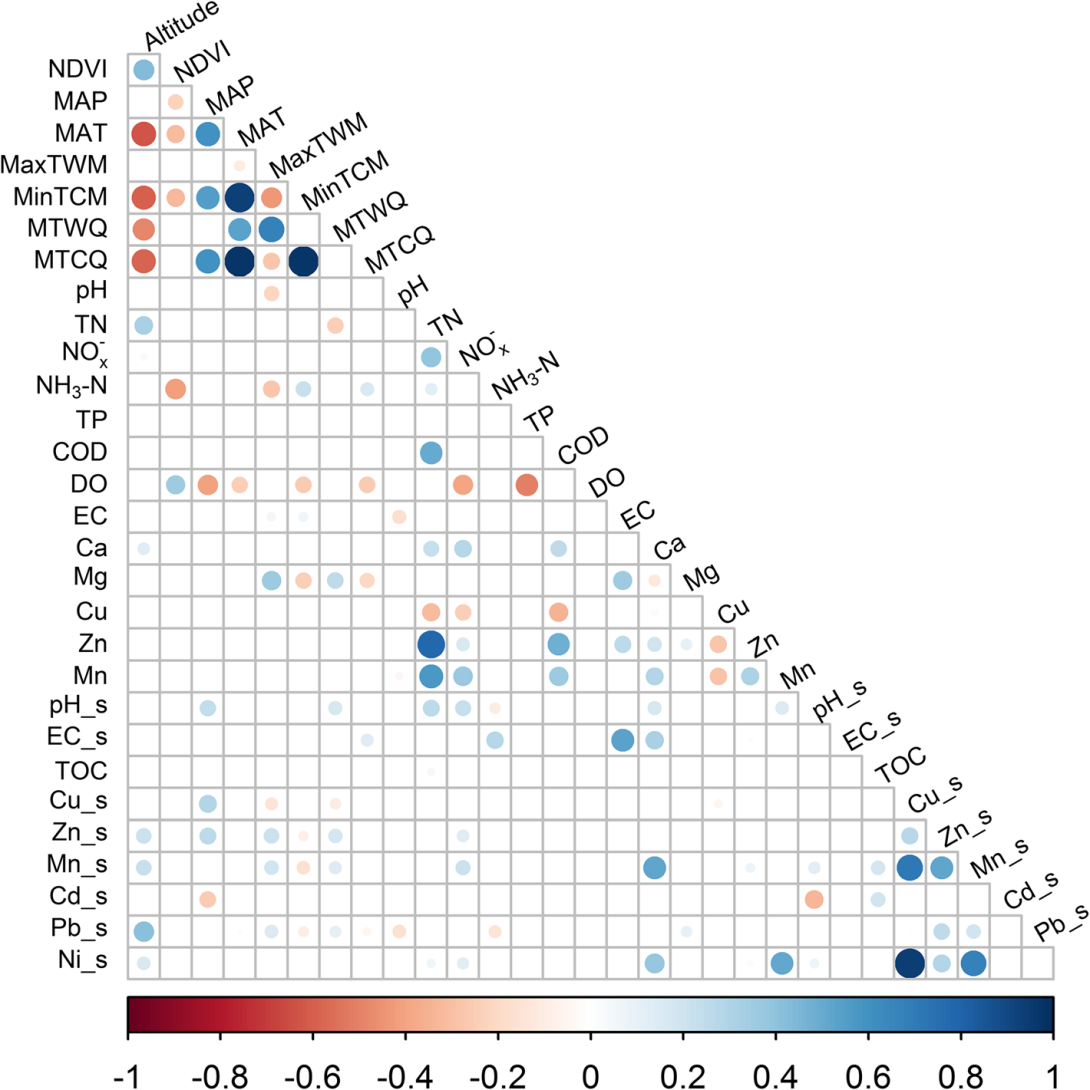
Spearman rank correlation matrix for environmental and physicochemical variables. The size of the circles indicates the magnitude of the correlation, the color represents the direction of the relationship and presence indicates *P* <  0.05. The suffixes, “_s”, indicate sediment measurements

Altitude showed a positive correlation with NDVI and negative correlations with climatic variables (MAT, MinTCM, MTWQ and MTCQ) except for MAP and MaxTWM (Fig. 3). NDVI showed significant negative correlation with MAT, MinTCM, and MTCQ. MAP was positively correlated with MAT, MinTCM, and MTCQ but was negatively correlated with DO. MaxTWM was negatively correlated with MinTCM and Mg. TN showed positive correlations with NO_x_^−^, COD, Zn and Mn. NH_3−_N was positively correlated with Ca. TP was negatively related to DO. Moreover, Cu_s showed significant positive correlations with Mn_s and Ni_s. Mn_s was also positively related to Ni_s.

### Differences between sites with and without *B. straminea*

Sites where *B. straminea* had higher altitude, mean annual precipitation, MinTCM and MTCQ, and lower MaxTWM and MTWQ (Table 3). TN, NO_x_^−^, NH_3−_N, Ca, Zn and Mn concentrations were higher in water samples with *B. straminea* snails, however, the DO and Mg were lower. For sediments, Zn was found to be higher at sites with snails (Table 3).

**Table 3:** Comparison of environmental and physicochemical characteristics between sites with and without *B. straminea*

	Presence of *B. straminea*		*P*-value
	No (*n* = 78)	Yes (*n* = 6)	
Landscape and climatic variables			
Altitude (m)	10.0 (4.0,33.2)	45.0 (43.5,53.2)	0.019
NDVI	0.7 (0.6,0.8)	0.7 (0.6,0.7)	0.951
MAP (mm)	1714 (1685,1818)	1969 (1937,2016)	0.004
MAT (°C)	22.2 (22.0,22.3)	22.2 (22.0,22.5)	0.807
MaxTWM (°C)	33.3 (32.9,33.8)	32.4 (31.8,32.5)	< 0.001
MinTCM (°C)	10.5 (9.8,11.4)	11.9 (11.7,12.1)	0.009
MTWQ (°C)	28.5 (28.2,28.6)	28.0 (27.8,28.3)	0.012
MTCQ (°C)	14.7 (14.1,15.2)	15.2 (15.1,15.5)	0.053
Water variables			
pH	6.71 (6.48, 7.05)	6.92 (6.52, 6.96)	0.889
TN (mg/L)	2.31 (1.27, 7.80)	8.33 (5.79, 12.9)	0.030
NO_x_^−^ (mg/L)	0.78 (0.29, 1.72)	3.10 (1.08, 4.53)	0.050
NH_3−_N (mg/L)	0.33 (0.10, 1.04)	2.28 (1.23, 5.90)	0.025
TP (mg/L)	0.13 (0.07, 0.20)	0.52 (0.17, 0.87)	0.071
COD (mg/L)	9.97 (4.48, 22.1)	14.1 (9.32, 19.1)	0.487
DO (mg/L)	5.64 (4.80, 6.51)	3.97 (3.62, 4.46)	0.039
EC (mS/m)	144 (69.4, 229)	92.0 (68.0, 113)	0.234
Ca (mg/L)	4.31 (1.01, 18.5)	29.2 (19.6, 104)	0.006
Mg (mg/L)	1.11 (0.76, 1.57)	0.02 (0.01, 0.53)	0.002
Zn (mg/L)	0.01 (0.00, 0.04)	0.08 (0.05, 0.19)	0.005
Mn (mg/L)	0.13 (0.05, 0.26)	0.32 (0.25, 0.61)	0.010
Sediment variables			
pH_s	6.98 (6.31, 7.31)	6.85 (6.66, 7.15)	1.000
EC_s (mS/m)	391 (211, 814)	962 (557, 1649)	0.124
TOC (g/kg)	27.4 (20.4, 37.8)	28.5 (20.3, 43.3)	0.907
Cu_s (mg/kg)	0.67 (0.13, 2.96)	2.84 (1.51, 22.5)	0.046
Zn_s (mg/kg)	5.76 (0.20, 22.4)	37.7 (22.3, 48.4)	0.005
Mn_s (mg/kg)	71.3 (0.57, 132)	199 (81.0, 212)	0.020
Cd_s (mg/kg)	0.13 (0.00, 0.19)	0.07 (0.04, 0.18)	0.820
Pb_s (mg/kg)	1.92 (0.04, 8.10)	2.76 (1.00, 4.81)	0.922
Ni_s (mg/kg)	1.25 (1.25, 1.25)	2.22 (1.25, 10.4)	0.099

Environmental and physicochemical variables are shown as median (25th and 75th percentile)

### Results of univariate and multivariate GLMM

Univariate GLMM found that presence of *B. straminea* was associated with NDVI (pMCMC = 0.007), MaxTWM (pMCMC = 0.024), water DO (pMCMC = 0.001), water Mg (pMCMC < 0.001) and sediment Log10(Zn_s) (pMCMC = 0.024) (Table 4). However, only MaxTWM (pMCMC < 0.001) and sediment Log10(Zn_s) (pMCMC < 0.001) were retained in the final model (Table 5).

**Table 4:** Estimates of univariate generalized linear mixed effects model

Variables	Posterior mean	95% Credible interval		pMCMC
		Lower	Upper	
Landscape and climatic variables				
Altitude (m)	0.04	−0.06	0.14	0.412
NDVI	42.37	7.80	86.28	0.007
MAP (mm)	0.02	−0.02	0.05	0.294
MAT (°C)	−1.41	−8.58	4.95	0.728
MaxTWM (°C)	−7.45	−17.35	−0.14	0.024
MinTCM (°C)	−0.01	−4.45	4.92	0.962
MTWQ (°C)	−5.00	−14.12	3.05	0.205
MTCQ (°C)	0.77	−4.40	6.68	0.734
Water variables				
pH	−2.52	−13.28	8.92	0.584
Log_10_(TN)	−3.12	−11.41	6.63	0.454
Log_10_(NO_x_^−^)	−3.14	−10.19	1.97	0.2.97
Log_10_(NH_3−_N)	0.59	−5.00	6.67	0.890
Log_10_(TP)	1.07	−2.46	4.44	0.522
Log_10_(COD)	0.41	−9.84	10.46	0.873
DO	3.89	0.59	7.56	0.001
Log_10_(EC)	0.26	−11.89	11.18	0.969
Log_10_(Ca)	3.75	−4.46	9.35	0.239
Mg	−23.69	−34.46	−12.69	< 0.001
Log_10_(Zn)	1.66	−4.94	7.94	0.598
Log_10_(Mn)	8.33	−3.81	21.57	0.182
Sediment variables				
pH_s	−4.08	−10.01	0.73	0.125
Log_10_(EC_s)	4.59	−5.13	16.16	0.380
Log_10_(TOC)	−0.10	−16.44	12.34	0.962
Log_10_(Cu_s)	−0.76	−2.06	3.72	0.577
Log_10_(Zn_s)	6.22	0.88	12.66	0.014
Log_10_(Mn_s)	0.68	−4.01	6.53	0.843
Log_10_(Cd_s)	0.79	−1.53	3.68	0.597
Log_10_(Pb_s)	−0.60	−2.68	1.23	0.555

**Table 5:** Results of the multivariate generalized linear mixed effects model

Variables	Posterior mean	95% Credible interval		pMCMC
		Lower	Upper	
Landscape and climatic variables				
MaxTWM (°C)	−18.75	−28.99	−6.77	< 0.001
Sediment variables				
Log_10_(Zn_s)	13.87	8.03	20.28	< 0.001

## Discussion

This study has been the most systematic and comprehensive attempt to elucidate the geographical distribution of *B. straminea* in mainland China. *B. straminea* snails had expanded their range from Shenzhen to its neighboring Dongguan and Huizhou, which was in agreement with previous observations [3, 8]. The existence of *B. straminea* is a prerequisite for the transmission of *S. mansoni* and has stirred up concerns about the outbreak of this disease in southern China. Schistosomiasis is an important travel-associated infection and frequently reported among returnees from endemic areas. In Europe, 1465 cases of imported schistosomiasis were reported between 1997 and 2010, 95% of which were acquired from the African continent [17]. An outbreak of urogenital schistosomiasis was reported in Corsica, France, where *Bulinus truncatus*, a compatible intermediate snail host for schistosome species in West Africa, were present [5]. Given the wide distribution of *B. truncatus* in southern Europe and the recent increase in migration from endemic areas, the risk of urogenital schistosomiasis has raised many concerns. Since the 1970s when China’s aid projects in Africa first started, population and goods movements have been on the increase. It was estimated that there are approximately 1 million Chinese living in Africa. Imported cases of *Schistosomiasis mansoni* or *haematobium* have been repeatedly reported among these returnees from African countries [4, 18]. People infected with African schistosomiasis can be misdiagnosed outside of endemic countries. There is also an increase of Africans coming to China for trade, education or travel. For instance, approximately 16 000 legal African residents lived in Guangzhou, a city near Shenzhen, in 2014 [18]. Their infection status remains largely unclear to this day. Although there is no record of *S. mansoni* transmission in China so far, the results of this study are informative for the efficient surveillance, control of the intermediate host and prevention of the introduction and transmission of a new species of *Schistosoma* in mainland China. Pre-travel health care education and post-travel consultations also proved useful for the prevention of schistosomiasis infection and early detection of asymptomatic infections [17].

*B. straminea* was first discovered in a stream in Hong Kong in 1974 [7] and in some ponds, ditches, and rivers in Shenzhen city, mainland China in 1981 [8]. More than thirty years later, this snail has just colonized water habitats in Shenzhen and nearby. High habitat suitability of *B. straminea* has been predicted in southern parts of Guangxi, Pearl River Delta areas of Guangdong, Hong Kong and restricted areas of North Taiwan [12, 19]. This prediction was only based on the distribution data in China and could be biased by the stage of invasion [20]. It is unknown how wide a geographical range this snail species may be able to colonize. In this respect, we compared the landscape and climatic characteristics between sites currently with and without *B. straminea*. Maximum temperature of the warmest month was found to be lower for locations where *B. straminea* was present. Air temperature has a direct impact on surface water temperature. Above-optimal water temperature can inhibit the fecundity and survival of adult snails and growth of juvenile ones [21]. Nevertheless, such unfavorable water temperature can be shunned by hiding under vegetation or moving deeper into the water [6, 21]. No differences in altitude, NDVI, precipitation and other temperature-related variables were found.

Within freshwater environments, physical and chemical properties of water and sediment are key factors for the survival of organisms [22, 23]. Type of water bodies and water quality have been suggested as important determinants influencing snail distribution [6]. *Biomphalaria* spp. abundance was found to be positively correlated with conductivity, hardness, calcium, nitrites plus nitrates, ammonium, and bicarbonates in rice fields in Argentina, but not with phosphates, pH or soil granulometry [24]. We found no significant differences between colonized and non-colonized areas in water pH, electrical conductivity, total nitrogen, nitrate and nitrites, ammoniacal nitrogen, total phosphorus, chemical oxygen demand and dissolved oxygen. There was no difference in sediment pH, electrical conductivity, and total organic carbon.

Metals also play an essential role in the survival, growth, and reproduction of *Biomphalaria* snails. Calcium has been associated with the growth of *B. glabrata* [25]. A high magnesium-to-calcium ratio was observed in streams where aquatic snails were absent [26]. Acute exposures to heavy metals (cadmium, lead and arsenic) were found to affect the reproduction of *B. glabrata* in terms of egg laying, hatching time and embryonic survival [27]. Low zinc concentrations were able to suppress the egg hatching, growth and sexual maturity of *B. glabrata* [28]. In the present study, none of the metals in the water differed between sites with and without *B. straminea*. Sediment zinc was higher at places with *B. straminea*. This phenomenon suggested that this snail has successfully adapted to urban water bodies polluted by industrial waste, domestic sewage, traffic and runoff.

This study has several limitations. *B. straminea* was identified using morphological straits, and we were unable to examine the phylogenetic relationships of *Biomphalaria* snail populations captured. Besides, the infection status of *S. mansoni* was not detected among the specimens. To date, there has been no evidence for *S. mansoni* in samples collected in Hong Kong, Shenzhen and Dongguan in the mainland China [10]. Both lab-reared and field-captured *B. straminea* snails from a stream in Luohu District of Shenzhen seemed incompatible with the *S. mansoni* Puerto Rican strain [29]. The compatibility between the snail vector *B. straminea* and *S. mansoni* varied among different geographical regions [29]. Further studies are warranted to confirm the compatibility between *B. straminea* snails sampled throughout the Zhujiang River Basin and *S. mansoni* strains from the other endemic regions. Additionally, since the malacological survey spanned over a year, seasonal variations of the water and sediment measurements were inevitable. To address the resultant seasonal effects, we included sampling month as a random effect term in the GLMM. Finally, other factors, including current velocity, the presence of predators and competing snails and microbial composition of the water were not analyzed in our study. Future survey should incorporate such measurements. It is worth mentioning that the presence or abundance of snails does not depend on a single environmental factor, but is rather the result of a complex interaction of multiple habitat factors [22]. Therefore, our results should be interpreted with caution.

## Conclusions

This study revealed the presence of *B. straminea* in Shenzhen and Dongguan of Guangdong Province, China. Significant differences were found in maximum temperature of the warmest month and sediment zinc between sites with and without *B. straminea*. Our results have important implications in prioritizing monitoring efforts to regions most at risk.

## Additional files

Additional file 1: Multilingual Abstracts in the five official languages of the United Nations. (PDF 475 kb)

Additional file 2: **Table S1.** Physicochemical parameters measured and relevant analytical methods in this study. (DOCX 24 kb)

## Abbreviations

Ca: Calcium
Cd: Cadmium
COD: Chemical oxygen demand
Cr: Chromium
Cu: Copper
CV: Coefficient of variation
DO: Dissolved oxygen
EC: Electrical conductivity
Fe: Iron
LOD: Limit of detection
Mg: Magnesium
Mn: Manganese
NDVI: Normalized difference vegetation index
NH_3−_N: Ammoniacal nitrogen
Ni: Nickel
NO_x-_: Nitrate and nitrites
Pb: Lead
SD: Standard deviation
TN: Total nitrogen
TOC: Total organic carbon
TP: Total phosphorus
Zn: Zinc
