# Supplementary material for: Spatial distribution and habitat suitability of Biomphalaria straminea, intermediate host of Schistosoma mansoni, in Guangdong, China
Source: Infect Dis Poverty. 2018 Nov 5;7:109. doi: 10.1186/s40249-018-0492-6 (PMC6217779; doi:10.1186/s40249-018-0492-6)
Supplement: Supplementary file 1 — Multilingual Abstracts in the five official languages of the United Nations. (PDF 475 kb) [file 40249_2018_492_MOESM1_ESM.pdf]

Translation of the abstract into the six official working languages of the United Nations

التوزّع المكاني والبيئة الملائمة *للقوقعة الزائفة ذات السرتين* العائل الوسيط *للبلهارسيا المنسونية* في مقاطعة يغوانغدونغ، الصين

Biao Zhou-Hui Deng, Yi-Wu Jiang, Zhuo-Yue Chen, Qing ,Quan Pei-Yu Huang, Fu-Ya Yang, Shao

نبذة مختصرة

معلومات أساسيّة: تعتبر القوقعة الزائفة ذات السرتينناقلًا شرسًا للمرض في الصين، مما يمثّل تهديدًا خطيرًا للصحة العامة. يُعدّ فهم العوامل المؤثرة على نشأة القوقعة من الأمور الحاسمة لتحسين قدرتنا على مجابهة الخطر المحتمل لانتشار وانتقال عدوى داء البلهارسيات. وسعَت تلك الدراسة إلى تحديد التوزّع المكاني للقوقعة الزائفة ذات السرتين في بر الصين الرئيس، وتحديد ما إذا كانت العوامل البيئية تختلف من مكان لآخر تبعًا لتواجد أو عدم تواجد القوقعة الزائفة ذات السرتين.

الاساليب: تم إجراء دراسة استقصائية على القوقعة الزائفة ذات السرتين بمقاطعة يغوانغدونغ، الصين. وتم تحديد القواقع باستخدام خصائصها التشريحية. أُخذت عينات من المياه والثفالة ، وتم تحليل الخصائص الفيزيائية لهما باستخدام المعايير القياسية الدولية. كما تم تسجيل المتغيرات الطبيعية والجوية لكل موقع. وقمنا بمقارنة الخصائص البيئية فيما بين المواقع سواء بتواجد أو عدم تواجد القوقعة الزائفة ذات السرتين *باستخدام* اختبار U مان - ويتني. واستخدمنا النماذج الخطية المعممة لحصر التأثيرات الموسمية.

النتائج: وُجدت القوقعة الزائفة ذات السرتين في ستة مواقع، منهم موقع في دونغجوان وخمسة مواقع في شنجن. وعثرت خريطة احتمالية عن بؤرة ساخنة للقوقعة الزائفة ذات السرتين منتشره في شنجن وهونج كونج. تم تصنيف المواقع التي استوطنتها القوقعة الزائفة ذات السرتينبأعلى متوسط مرتفع، وهو يعني هطول أمطار سنوية ودرجة حرارة معتدلة. تحتوى المياة المصابة بالقواقع على أعلى متوسط تركيز في مجموع كل من النيتروجين والنترات والنيتروجين الأمونياكي والزنك والمنغنيز، لكن القليل من الأكسجين والماغنسيوم المذابين. تحتوي الثُفَالَة المصابة بالقواقع على أعلى متوسط تركيز لكل من الحديد والزنك والمنغنيز. وقد ارتبطت *القوقعة* الزائفة ذات السرتين بدرجة الحرارة القصوى لأكثر الشهور دفئًا(pMCMC < 0.001) وثفالة الزنك(pMCMC < 0.001).

الاستنتاجات : القوقعة الزائفة ذات السرتين تنتشر في شنجن والمناطق المحيطة بها في غواندونغ بالصين. تختلف المواقع المتواجد فيها *القوقعة الزائفة ذات السرتين* عن تلك المواقع التي لاتتواجد فيها من حيث درجة الحرارة القصوى لأكثر الشهور دفئًا وثفالة الزنك. وينبغي أن تستمر المراقبة لرصدّ انتشار هذة القوقعة في الصين.

Translated from English version into Arabic by Mohamed Fouad, proofread by Mais Salsa, through

TRANSLATORS
WITHOUT BORDERS

曼氏血吸虫病中间宿主藁杆双脐螺在中国广东省的空间分布和栖息地适宜性

杨亚，黄少玉，裴福全，Chen Yue，姜庆五，邓卓晖，周艺彪

摘要

背景：在中国，藁杆双脐螺是一种入侵生物媒介，对公众健康构成重大威胁。研究影响这种螺生存的相关因素，对于提高管控藁杆双脐螺扩散和曼氏血吸虫病潜在传播风险的能力至关重要。本研究旨在确定藁杆双脐螺在中国大陆的空间分布及有螺和无螺地区之间的环境因素是否存在差异。

方法：在中国广东省开展了藁杆双脐螺分布的现场调查。螺类的鉴别是基于解剖学特征。在调查点采取了水和沉积物样本，通过国家标准方法对样本的理化特征进行检测。同时收集了每个采样点的景观和气候变量。我们使用 Mann-Whitney U 检验比较了有螺和无螺采样点之间的环境因素之间的差异。使用广义线性混合模型来校正季节的影响。

结果：在 6 个地点发现了藁杆双脐螺分布，其中 1 个点位于东莞，其他 5 个点均位于深圳。分布概率图显示在深圳和香港是藁杆双脐螺分布的热点。藁杆双脐螺分布点的高程和年平均降水量的中位数更高，温度更为温和。有藁杆双脐螺分布的水样在总氮、硝酸盐、亚硝酸盐、氨氮、钙、锌和锰的中位浓度更高，但是溶解氧和镁的中位浓度较低。有藁杆双脐螺分布的沉积物中铜、锌和锰的中位浓度较高。藁杆双脐螺分布与最热月的最高温度（pMCMC <0.001）和沉积物中的锌（pMCMC <0.001）相关。
结论：藁杆双脐螺分布于中国广东省的深圳及其周边地区。有螺和无螺区在最热月的最高温度和沉积物锌浓度方面存在差异。应继续监测藁杆双脐螺在中国的扩散情况。

Translated from English version into Chinese by Ya Yang

La répartition dans l'espace et les lieux de propagation de *Biomphalaria straminea* , hôte intermédiaire de *Schistosoma mansoni,* à Canton, en Chine.

Ya Yang, Shao-Yu Huang, Fu-Quan Pei, Yue Chen, Qing-Wu Jiang, Zhuo-Hui Deng, Yi-Biao Zhou

Résumé
Contexte: *Biomphalaria straminea* est un vecteur invasif en Chine qui représente une menace sérieuse de santé publique. Il est essentiel de comprendre les facteurs qui régissent la propagation de cet escargot pour améliorer notre capacité à gérer sa diffusion et le risque potentiel de transmission de schistosomiasie. Cette étude cherche à déterminer la répartition dans l'espace de *B. straminea* en Chine continentale, et si les facteurs environnementaux varient selon les endroits infectés ou non par *B. straminea* .
Méthodes: Une étude malacologique de *B. straminea* a été menée dans la province de Canton en Chine. Les escargots ont été identifiés en utilisant des caractéristiques anatomiques. Des échantillons d'eau et de sol ont été pris et leurs propriétés physiques et chimiques ont été analysées en utilisant les méthodes standard au niveau national. Des données climatiques et géographiques ont également été enregistrées pour chaque lieu. Nous avons comparé les caractéristiques environnementales entre tous les endroits infectés ou non par *B. straminea* en utilisantle test Mann-Whitney U. Ensuite, nous avons utilisé les modèles linéaires mixtes généralisés pour nous rendre compte des effets saisonniers.
Résultats: *B. straminea* a été localisée dans six endroits, dont un à Dongguan et cinq à Shenzhen. Acartographie des foyers probables a trouvé un foyer actif deB. straminea à Shenzhen et à Hong Kong. Les endroits infectés par *B. straminea* étaient caractérisés par une altitude médiane élevée, des précipitations annuelles moyennes et une température modérée. L'eau contenant les escargots avait une concentration médiane totale de nitrogène, de nitrate et de nitrite, de nitrogène ammoniacal, de calcium, de zinc et de manganèse plus haute, mais des taux d'oxygène soluble et de magnésium plus bas. Les sols contenant les escargots avaient des taux moyens de cuivre, de zinc et de manganèse plus hauts. *B. straminea*était associée à température maximale correspondant au mois le plus chaud (pMCMC < 0.001) et au taux de zinc présent dans le sol le plus haut (pMCMC < 0.001).
Conclusions: *B. straminea* est présente à Shenzhen et dans ses zones avoisinantes à Canton, Chine. Les endroits infectés ou non par *B. straminea* varient selon la température maximale du mois le plus chaud et selon le taux de zinc des sols. La surveillance devrait être maintenue pour gérer la propagation de cet escargot en Chine.

Translated from English version into French byClaire Vizy, proofread by Gram Matike, through

Пространственное распределение и устойчивость среды обитания*биомфалярии (рода пресноводных улиток),* являющейся промежуточным хозяином *кишечного шистосомоза,* в Гуандуне – провинции Китая.

Авторы: Я Ян, Шао-Юй Хуан, Фу Цюань Пэй, Юэ Чэнь, Цин-У Цзян, Чжо-Хой Дэн, И-Бяо Чжоу.

Краткий обзор

Справочная информация: *Биомфалярия* – это инвазивный переносчик инфекции в Китае, представляющий собой серьёзную угрозу местному населению. Определение факторов, влияющих на резкий рост численности улиток, существенно поможет сократить их количество и свести к минимуму риск передачи шистосомоза. Данное исследование позволит выявить пространственное распределение *биомфалярии* на территории материкового Китая, а также определить существует ли разница экологических факторов в заражённых и не заражённых *зонах.*
Методы: Малакологи провели исследование *биомфалярии* в Гуандуне. Род улиток был определён с помощью биологической систематики. Затем специалисты проанализировали физико-химические свойства проб воды и осадочного слоя, используя стандартные методы. Рельеф местности и её климатические изменения тоже были приняты во внимание. Благодаря U-критерию Манна-Уитни, мы сравнили экологические характеристики *заражённых* и не заражённых зон. Далее мы использовали обобщённые линейные модели, чтобы продемонстрировать сезонные колебания.
Результаты: *исследования: Наибольшая* численность биомфалярии была обнаружена в шести районах: один из них находится в Дунгуане, а остальные в Шэньчжэне. Прогностическая карта выявила наиболее активное *распространение* биомфалярии в Шэньчжене и Гонконге. Места скопления *биомфалярии* отличались большей высотой над уровнем моря, средним количеством годовых осадков и умеренной температурой. Вода, в которой обитали улитки, содержит больше средних концентраций общего азота, нитратов и нитритов, аммиачного азота,кальция, цинка и марганца. Однако в ней присутствует меньше растворенного кислорода и магния. В осадочном слое с улитками более высокая концентрация меди, цинка и марганца. *Появление биомфалярии* связано с максимальной температурой в самый теплый месяц ($pMCMC < 0.001$) и с накоплениями цинка в почве ($pMCMC < 0.001$).
Вывод: *Биомфалярия* активно распространяется в Шэньчжене и соседних районах китайской провинции Гуандун. Заражённые *биомфалярией* местности отличаются от не заражённых максимальной температурой в самый тёплый месяц и количеством накоплений цинка в почве. Необходимо дальнейшее наблюдение за размножением этого вида улиток в Китае.

Translated from English version into Russian by Lamiya Mehtieva, proofread by Tatyana Ven, through

Distribución espacial e idoneidad del hábitat de *Biomphalaria straminea*, huésped intermediario de *Schistosoma mansoni*, en Guangdong, China

Ya Yang, Shao-Yu Huang, Fu-Quan Pei, Yue Chen, Qing-Wu Jiang, Zhuo-Hui Deng, Yi-Biao Zhou

Resumen

Antecedentes: *Biomphalaria straminea* es un vector invasivo en China que representa una amenaza importante para la salud pública. Entender los factores que afectan el establecimiento de este caracol es crítico para la mejora de nuestra capacidad de gestionar su propagación, así como el posible riesgo de transmisión de la esquistosomiasis. Este estudio buscaba determinar la distribución espacial de *B. straminea* en China Continental y averiguar si existían divergencias en los factores medioambientales entre los lugares con y sin presencia de *B. straminea*.

Metodología: El estudio malacológico de *B. straminea* se llevó a cabo en la provincia de Guangdong en China. Los caracoles se identificaron mediante el uso de claves anatómicas. Se tomaron muestras de agua y de sedimento y se analizaron sus propiedades fisicoquímicas a través de los métodos estándar nacionales. Adicionalmente, se recogieron variables acerca del paisaje y del clima para cada lugar. Comparamos las características medioambientales entre emplazamientos con y sin *B. straminea* utilizando la prueba U de Mann-Whitney. Además empleamos modelos lineales generalizados mixtos con el fin de tener en cuenta los efectos estacionales.

Resultados: *B. straminea* fue encontrada en seis lugares, incluidos uno en Dongguan y cinco en Shenzhen. El mapa de probabilidad encontró un punto de alta concentración en la *distribución de* B. straminea en Shenzhen y en Hong Kong. Los lugares ocupados por *B. straminea* se caracterizaban por niveles más elevados de altitud mediana, precipitación anual media y temperatura moderada. El agua que tenía caracoles presentaba una mayor concentración mediana de nitrógeno total, nitrato y nitritos, nitrógeno amoniacal, calcio, zinc y manganeso, pero tenía menos oxígeno disuelto y magnesio. Los sedimentos que contenían caracoles mostraban niveles medianos más altos de cobre, zinc y manganeso. *B. straminea* se relacionó con la temperatura máxima del mes más cálido (pMCMC < 0.001) y del zinc en el sedimento (pMCMC < 0.001).

Conclusiones: *B. straminea* está repartida por Shenzhen y sus alrededores en Guangdong, China. Los lugares con y sin *B. straminea* diferían en la temperatura máxima del mes más cálido y del zinc en el sedimento. El seguimiento debe continuarse con el objetivo de supervisar la propagación de este caracol en China.

Translated from English version into Spanish by Jennifer M Lang, proofread by Sara Fairen, through

TRANSLATORS
WITHOUT BORDERS
