# Supplementary material for: Spatial distribution and habitat suitability of Biomphalaria straminea, intermediate host of Schistosoma mansoni, in Guangdong, China
Source: Infect Dis Poverty. 2018 Nov 5;7:109. doi: 10.1186/s40249-018-0492-6 (PMC6217779; doi:10.1186/s40249-018-0492-6)
Supplement: Supplementary file 2 — Table S1. Physicochemical parameters measured and relevant analytical methods in this study. (DOCX 24 kb) [file 40249_2018_492_MOESM2_ESM.docx]

Supplementary to “**Spatial distribution of Biomphalaria straminea in relation to environmental factors in China”**

Ya Yang^1,*^, Shaoyu Huang^2,*^, Fuquan Pei^2^, Qingwu Jiang^1^, Zhuohui Deng^2,†^, Yibiao Zhou^1,†^

^1^Key Laboratory of Public Health Safety, Ministry of Education, Tropical Disease Research Center, Department of Epidemiology, School of Public Health, Fudan University, Shanghai, China

^2^Guangdong Provincial Center for Disease Control and Prevention, WHO Collaborating Centre for Surveillance, Research and Training of Emerging Infectious Diseases, Guangzhou, Guangdong, China

^*^These authors contributed equally to this work.

^†^Correspondence: tracydzh@163.com; z_yibiao@hotmail.com

## Table S1 Physicochemical parameters measured and relevant analytical methods in this study.

| **Type of sample** | **Variables (abbreviation)** | **Detection Methods (Instruments)** | **Limit of detection (LOD)** |
| --- | --- | --- | --- |
| **Water** | Total nitrogen (TN) | Alkaline potassium persulfate ultraviolet spectrophotometry (UV759, Shanghai INESA Scientific Instrument Co., Ltd., China) | 0.05 mg/L |
|  | Nitrate and nitrites (NO_x-_) | Ultraviolet spectrophotometry (UV759, Shanghai INESA Scientific Instrument Co., Ltd., China) | 0.08 mg/L |
|  | Ammonia nitrogen (NH_3-_N) | Nessler’s reagent spectrophotometry (UV759, Shanghai INESA Scientific Instrument Co., Ltd., China) | 0.025 mg/L |
|  | Total phosphorus (TP) | Ammonium molybdate spectrophotometry (UV759, Shanghai INESA Scientific Instrument Co., Ltd., China) | 0.01 mg/L |
|  | Chemical Oxygen Demand (COD) | Rapid digestion spectrophotometry (COD digestion instrument XJ-III, Tomorrow environmental protection instrument Co., Ltd., China) | 2 mg/L |
|  | Dissolved Oxygen (DO) | Dissolved oxygen analyzer(JPBJ-608, Shanghai INESA Scientific Instrument Co., Ltd., China) |  |
|  | pH | pH meter (pH meter, OHAUS (Changzhou) Co., Ltd., China ) | 2-12（0-40℃） |
|  | [Electrical conductivity](http://www.baidu.com/link?url=KZrgFynhPbZfsGEn5VxIGR5S6ntnLpYR-zZ-GZJIDZHCPYa9mieMYGBOVtIYki40mwtoNRAWyBdAi8yzRiSNb3xTzeLDJ6gniQ5gDze-U47) (EC) | Electrical conductivity analyzer (DDS-307A, , Shanghai INESA Scientific Instrument Co., Ltd., China) | 0-500 mS/m（0-40℃） |
|  | Calcium (Ca) | Atomic absorption spectrometry (ZA3300, Hitachi, Japan) | 0.02 mg/L |
|  | Magnesium (Mg) |  | 0.02 mg/L |
|  | Copper (Cu) |  | 0.04 mg/L |
|  | Zinc (Zn) |  | 0.009 mg/L |
|  | Iron (Fe) |  | 0.01 mg/L |
|  | Manganese (Mn) |  | 0.01 mg/L |
|  | Cadmium (Cd) |  | 0.05 mg/L |
|  | Lead (Pb) |  | 0.1 mg/L |
|  | Chromium (Cr) |  | 0.03 mg/L |
|  | Nickel (Ni) |  | 0.02 mg/L |
| Sediment | pH_s | pH meter (pH meter, OHAUS (Changzhou) Co., Ltd., China | 2-12（0-40℃） |
|  | [Electrical conductivity](http://www.baidu.com/link?url=KZrgFynhPbZfsGEn5VxIGR5S6ntnLpYR-zZ-GZJIDZHCPYa9mieMYGBOVtIYki40mwtoNRAWyBdAi8yzRiSNb3xTzeLDJ6gniQ5gDze-U47) (EC_s) | Electrical conductivity analyzer (DDS-307A, , Shanghai INESA Scientific Instrument Co., Ltd., China) | 0-500 mS/m（0-40℃） |
|  | Total organic carbon (TOC) | Potassium dichromate oxidation spectrophotometry (UV759, Shanghai Jingke Industrial Co., Ltd., China) | 0.6g/Kg |
|  | Copper (Cu_s) | Atomic absorption spectrometry (ZA3300, Hitachi, Japan) | 0.1 mg/Kg |
|  | Zinc (Zn_s) |  | 0.4 mg/Kg |
|  | Iron (Fe_s) |  | 100 mg/L |
|  | Manganese (Mn_s) |  | 0.1 mg/Kg |
|  | Cadmium (Cd_s) |  | 0.0001 mg/Kg |
|  | Lead (Pb_s) |  | 0.0005 mg/Kg |
|  | Chromium (Cr_s) |  | 2.5 mg/Kg |
|  | Nickel (Ni_s) |  | 2.5 mg/Kg |
